# Andrographolide inhibits human serum albumin fibril formations through site-specific molecular interactions

**DOI:** 10.1039/c8ra04637a

**Published:** 2018-08-31

**Authors:** Aalok Basu, Sagar Bhayye, Sonia Kundu, Aatryee Das, Arup Mukherjee

**Affiliations:** Division of Pharmaceutical and Fine Chemical Technology, Department of Chemical Technology, University of Calcutta 92 A.P.C. Road Kolkata 700009 West Bengal India arupm1234@gmail.com +91 33 23519755 +91 33 23508387; Dr. B.C. Roy College of Pharmacy and Allied Health Sciences Bidhannagar Durgapur 713206 West Bengal India

## Abstract

Protein misfolding and fibrillation are the fundamental traits in degenerative diseases like Alzheimer's, Parkinsonism, and diabetes mellitus. Bioactives such as flavonoids and terpenoids from plant sources are known to express protective effects against an array of diseases including diabetes, Alzheimer's and obesity. Andrographolide (AG), a labdane diterpenoid is prescribed widely in the Indian and Chinese health care systems for classical efficacy against a number of degenerative diseases. This work presents an in depth study on the effects of AG on protein fibrillating pathophysiology. Thioflavin T fluorescence spectroscopy and DLS results indicated concentration dependent inhibition of human serum albumin (HSA) fibrillation. The results were confirmed by electron microscopy studies. HSA fibril formations were markedly reduced in the presence of AG. Fluorescence studies and UV-Vis experiments confirmed further that AG molecularly interacts with HSA at site. *In silico* molecular docking studies revealed hydrogen bonding and hydrophobic interactions with HSA in the native state. Thus AG interacts with HSA, stabilizes the native protein structure and inhibits fibrillation. The results demonstrated that the compound possesses anti-amyloidogenic properties and can be promising against some human degenerative diseases.

## Abstract 2

Andrographolide inhibited HSA protein fibrillation through site specific interactions.

## Graphical abstract

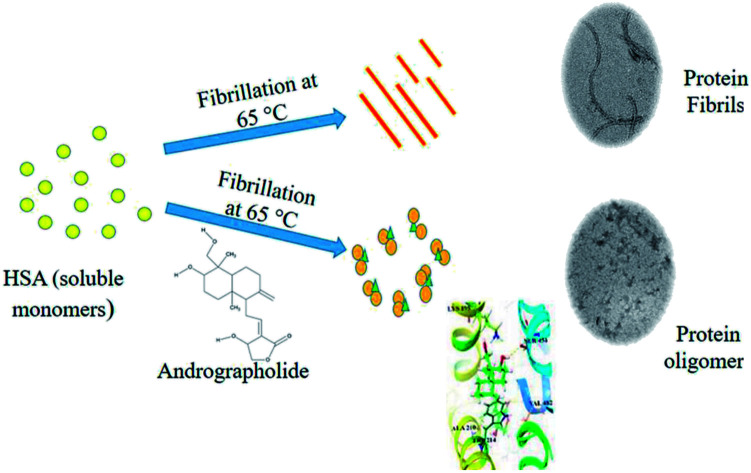

## 1. Introduction

Proteins are complex yet exquisitely arranged molecules which carry out well defined biological functions. Three dimensional macromolecule structure and spatial arrangement of functional groups are major determinants for protein bio-functions. Physiological stress conditions such as oxidative imbalances, enhanced cellular degradation or dominant negative mutation often initiate a biochemical cascade. This leads to molecular self-association of proteins which can result in amyloidogenesis. Self-associated fibrils of α-synuclein, HSA and insulin are predominantly cytotoxic.^1–3^ Similar molecular disposition brings about cell death and persistent physiological abnormalities.^4^ Protein misfolding and fibrillation are some of the underlying causes in a number of degenerative conditions such as Alzheimer's disease, parkinsonism, type 2 diabetes, atherosclerosis and others.^5^ Therapeutic stratagems to mitigate or prevent amyloidogenic diseases include stabilization of native protein structures and increased clearance of already misfolded protein aggregates.^6,7^

Different bioactive principles derived from natural sources have been explored recently as fibrillation inhibitors.^8^ Andrographolide (AG) is a labdane diterpenoid extracted from *Andrographis paniculata* Nees herb. AG has been traditionally used in Indian and Chinese health care systems for contending a myriad of ailments and degenerative diseases. AG demonstrated pronounced efficacy as hepatoprotective, antimalarial, antitumor agent, in cognitive improvement, immunomodulation, and several others.^9–13^ AG is an effective molecule in a multitude of amyloidogenic diseases such as diabetes, rheumatoid arthritis, and Aβ neurotoxicity.^14–16^ A comprehensive molecular mechanism underlying the therapeutic effects of AG has not yet been explored.

In the present work, we have studied the effect of different concentrations of andrographolide on HSA fibrillation at an elevated temperature. The process of fibrillation has been monitored using a combination of dynamic light scattering techniques, Thioflavin T fluorescence spectroscopy and electron microscopy. Further efforts have been made to interpret the interactions between andrographolide and HSA protein through multi-spectroscopic and *in silico* molecular docking techniques. This study reveals an insight into the protective mechanism of AG against amyloid formations and protein misfolding disease conditions.

Human serum albumin (HSA) is the most abundant, soluble, single chained protein containing 585 amino acids. The protein remains stabilized by 17 disulfide bridges in heart-like self-assembly formations. It contributes to storage and transportation of endogenous and exogenous substances such as hormones, fatty acids, nutrients and drugs.^17^ Isolated from the plasma, HSA has been extensively used as a model α-helical protein to explore the mechanisms of protein–ligand bindings,^18–21^ protein misfolding and fibrillation.^22–24^ The aggregation of albumin protein progresses through partial misfolding of its native structure and the process can be replicated *in vitro* using various conditions such as elevated temperature, metal ions, treatment with denaturing agents like alcohol, and lowering of pH.^25,26^ A number of small molecular drugs has been observed to bind and resist conformational changes of the similar tertiary protein structures.^27^

## 2. Experimental

### 2.1 Materials

AG was obtained from Natural Remedies (Bangalore, India). HSA (human serum albumin) was purchased from Sigma Aldrich, St. Louis, US. ThT (Thioflavin T) dye used for fibrillation experiments was purchased from TCI chemicals, Japan. Other chemicals and solvents were procured from Merck, India. All solutions were prepared using Milli-Q water from laboratory purification system, unless otherwise mentioned.

### 2.2 Protein solution

A stock solution of HSA (100 μM) was prepared by dissolving lyophilized HSA in buffer solution (10 mM PBS, pH 7.4), and its concentration was determined spectrophotometrically using a molar extinction coefficient of 35 219 M^−1^ cm^−1^ at 280 nm. The solution was filtered using Millipore filter (Millipore, USA) of pore size 0.44 μm.

### 2.3 Measurement of fibril formation

HSA solutions with or without AG were incubated at 65 ± 2 °C in a reciprocating shaker bath set at 80 rpm to induce amyloid fibril formation. The formation of HSA fibrils in presence of different ratios of AG (1 : 0, 1 : 0.5, 1 : 1 and 1 : 2) was monitored by DLS technique performed on a Malvern Nano ZS zetasizer (Malvern Instruments, UK) against a 4 mW He–Ne laser beam, 633 nm, having a back scattering angle of 173°. The HSA fibrillar samples were diluted to 2 μM in Milli-Q water and the average radius was recorded after 15 runs for each sample at 25 °C. The dynamic information of the aggregates present in protein solution was analyzed from an intensity autocorrelation function based on translational diffusion co-efficient, using the following equation:

1 *R*_h_ = *kT*/6π*ηD*_w_

where *R*_h_ is the hydrodynamic radius in nm, *k* is Boltzmann's constant, *T* is absolute temperature in kelvin, *η* is the viscosity of the medium and *D*_w_ is the translational diffusion co-efficient at 25 °C.

The fibrillation process was more intently studied through ThT fluorescence spectroscopy. HSA samples (10 μM) were co-incubated with ThT dye solutions (2 μM) at room temperature.^28^ The fluorescence intensity was measured using a spectrofluorimeter (Perkin-Elmer LS-5, PerkinElmer Inc., US), having exciting and emission wavelengths set at 450 and 482 nm. All blanks, native HSA and AG were initially checked to confirm non-responsiveness to ThT fluorescence.

### 2.4 HSA AG interaction study

The UV-visible absorption spectra of HSA (5 μM) in the absence and presence of varying concentrations (0–6 μM) of AG were recorded on UV-Vis spectrophotometer UV-1800 (Shimadzu Corp., Japan) using matched quartz cuvettes of 1.0 cm path length. The solutions were allowed to incubate at 37 °C for 30 min and the spectra were then obtained in the scanning range of 250–350 nm.

Fluorescence emission spectra were recorded from 300 nm to 500 nm with an excitation wavelength of 280 nm using a fluorescence spectrofluorimeter. The instrument was equipped with a water bath circulator controlled by Neslab RTE 100 thermostat. A fixed concentration of HSA (5 μM) was titrated with varying concentration of AG (0–6 μM) and a fluorescence quenching spectra was obtained at 37 °C. It was also confirmed that AG alone did not exhibit fluorescence on excitation at 280 nm. All experiments were carried out in triplicates to reduce noise occurrence.

### 2.5 Molecular modeling study

The structure of AG was prepared in LigPrep.^29^ The induced fit docking (IFD) protocol used in this study was carried out in three consecutive steps.^30^ First, the ligand was docked into a rigid receptor model with scaled-down van der Waals (vdW) radii. A vdW scaling of 0.5 was used for both the protein and ligand nonpolar atoms. A constrained energy minimization was carried out on the protein structure, keeping it close to the original crystal structure while removing bad steric contacts. Energy minimization was carried out using the OPLS2005 force field with implicit solvation model until the default criteria were met. The centroid of the co-crystal ligand was used to define the location of the binding site and the dimension of the energy grids for the initial docking was set to 25 Å. The Glide XP mode was used for the initial docking, and 20 ligand poses were retained for protein structural refinements.^31^ In the second step, prime was used to generate the induced-fit protein–ligand complexes. Each of the 20 structures from the previous step was subjected to side-chain and backbone refinements. All residues with at least one atom located within 5.0 Å of each corresponding ligand pose were included in the prime refinement. The refined complexes were ranked by prime energy, and the receptor structures within 30 kcal mol^−1^ of the minimum energy structure were passed through for a final round of Glide docking and scoring. In the final step, each ligand was re-docked into every refined low-energy receptor structure produced in the second step using Glide XP at default settings. Induced fit docking was performed using IFD docking module of Schrödinger software package software. Best dock pose of AG selected based on interaction shown as well as Glide XP and IFD score.

### 2.6 Transmission electron microscopy

Transmission electron microscopy was performed to analyze the size and structural attributes of the HSA fibrils formed in presence and absence of AG were incubated over a period of 6 days. 10 μL of the sample placed on a carbon coated copper grid (TED PELLA) and negatively stained with freshly prepared 2% uranyl acetate solution. Electron micrographs were obtained from an electron microscope (JEOL JEM 2100, Japan) operated at 120 kV.

### 2.7 Circular dichroism

Far UV-CD spectra were recorded between 190 to 250 nm on a JASCO J-815 CD spectro-polarimeter set with a scan speed of 100 nm min^−1^ and bandwidth of 1 nm. Samples (2.3) were placed in a 1 mm cell and measurements were taken at 25 °C. Each spectrum was baseline corrected and ellipticity was recorded from an average of three scans acquired.

### 2.8 Statistical analysis

All the experiments were performed in triplicates and the data have been presented as mean ± S.D.

## 3. Results and discussion

### 3.1 Effect of AG on HSA fibril formation

**Fig. 1:**
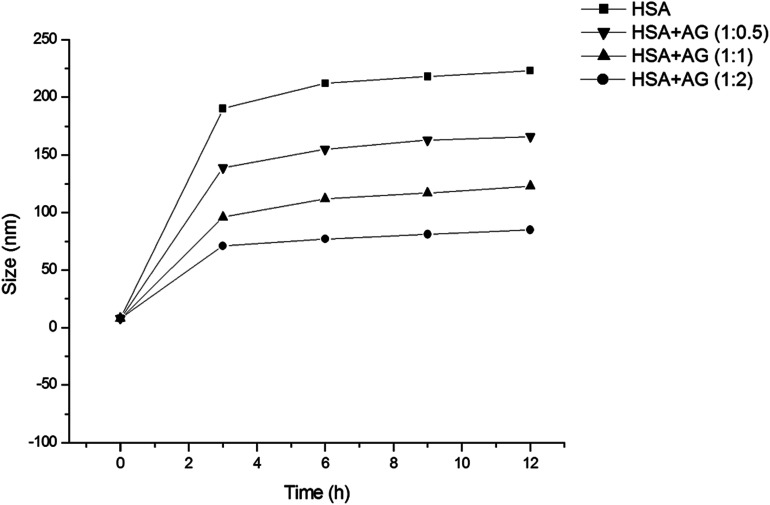
Fibril growth of HSA in absence and presence of different ratios of AG as measured by DLS at 25 °C.

HSA has been used as a model protein in amyloid research and that can form fibrils at elevated temperature and agitations.^24,32^ HSA was co-incubated with or without AG at 65 ± 2 °C and DLS studies were conducted in order to measure the hydrodynamic radii of HSA aggregates throughout the incubation phases (Fig. 1). The samples were appropriately diluted to diminish the protein–protein interaction and viscosity effects, which are imminent at higher concentrations.^33^*R*_h_ of untreated HSA was found to be 4.1 ± 0.3 nm, and that was similar to earlier works.^34^ When incubate at 65 °C, that value escalated sharply up to 218 ± 4.2 nm, signifying partial unfolding of albumin tertiary structure^35^ and generation of high molecular protein aggregates. However, the growth of protein aggregates treated with AG in ratios of 1 : 0.5, 1 : 1, and 1 : 2 were restricted to values of 166 ± 3.7 nm, 123 ± 3.1 nm and 85 ± 2.8 nm, respectively within 4 h of incubation and remained unaltered for the rest of the incubation period. The data obtained from DLS studies highlighted concentration dependent changes in the hydrodynamic radii of HSA protein when co-incubated with AG. This could be a result of unfolding of polypeptide chains, or conformational shifts due to oxidation/reduction state, or formation of AG–HSA complexes.^36^

**Fig. 2:**
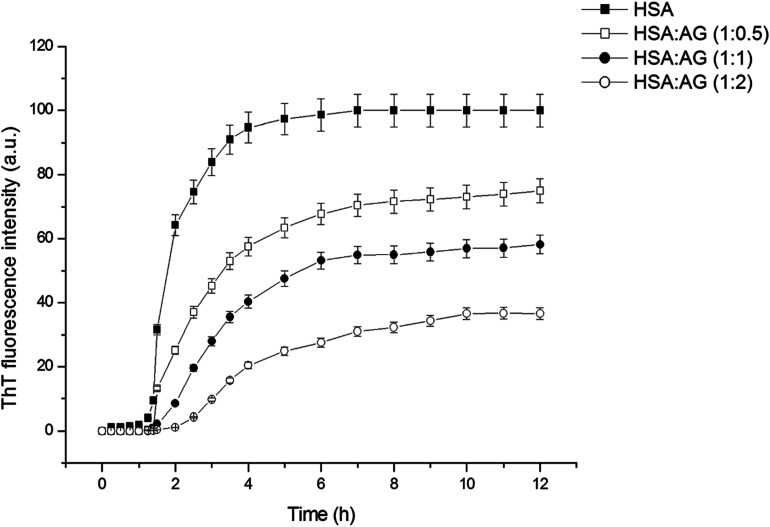
ThT growth curve of HSA in absence and presence of different ratios of AG.

ThT is an extrinsic fluorescent dye used routinely as a characteristic molecular probe for monitoring of amyloid formations.^37^ ThT intensity of untreated HSA increased progressively after a lag period of 1 h and reached saturation at 6 h (Fig. 2). This was in agreement with amyloidogenic proteins polymerization models.^4^ However, a concentration dependent inhibitory effect was observed when HSA was incubated with AG in different stoichiometric ratios. The fall of ThT intensity at saturation phase was evidenced at higher ratios of 1 : 2::HSA : AG as compared to a sub-stoichiometric ratio of 1 : 0.5 for HSA : AG. The data were also fitted using the following sigmoidal equation (Microcal Origin 6.0) to evaluate lag-time information.2
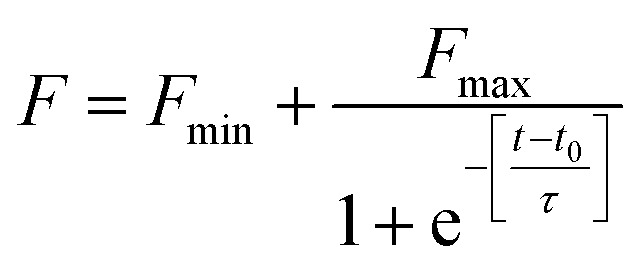
where *Y* is the ThT fluorescence intensity, *F*_min_ is minimal ThT fluorescence intensity, *F*_max_ is the maximum ThT fluorescence intensity, *t* is incubation time, *t*_0_ is the time required to attain 50% of maximal fluorescence. The apparent first order rate constant for protein fibril growth is expressed as 1/*τ* and its lag time is calculated as *t*_0_ − 2*τ*. The analysis (Table 1) revealed that AG increased the lag time for HSA fibrillation and subdued the rate of polymerization in a concentration dependent manner. This indicates that AG delayed the aggregation of HSA monomers and subsequent primary nuclei formation.

**Table 1:** Lag time and apparent rate constant of HSA fibrillation

HSA : AG	Lag time (h)	Apparent rate constant (h^−1^)
1 : 0	0.68 ± 0.066	2.68
1 : 0.5	0.88 ± 0.132	1.24
1 : 1	1.37 ± 0.081	1.21
1 : 2	1.44 ± 0.158	0.86

### 3.2 Characterization of binding interaction between HSA and AG

**Fig. 3:**
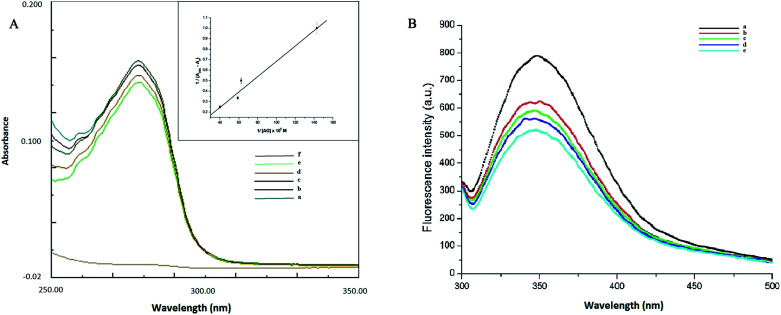
(A) Absorption spectra of AG (a), HSA in absence (b) and presence of different concentration of AG (c–f) (B) fluorescence spectra of HSA in presence of different concentration of AG (a–e).

Understanding the interactions between HSA globular structure and small molecules is perceived as one important step to gain insight into protein fibrillation inhibition mechanisms.^7^ UV absorption spectroscopy is an effective tool to detect the serum albumin structural changes and specific interactions with different molecular species.^38^Fig. 3A(b) shows the absorption spectra of HSA featuring a distinctive peak at 278 nm due to presence of amino acid residues such as phenylalanine, tryptophan and tyrosine. It was further confirmed that AG did not exhibit any significant absorbance in that region. Change of UV absorbance intensity was recorded with increasing concentration of AG (c–f) incubation. This suggested non-covalent interactions between HSA and AG. Such interactions were likely due to hydrophobic attachments of AG with phenyl ring of aromatic amino acids present in HSA binding cavities. These observations primarily indicated perturbation in the microenvironment during ground-state complex formation.^39,40^ The binding constant value (*K*) was obtained from the method as described by Benesi and Hildebrand,^41^ using a double reciprocal plot based on the equation:3
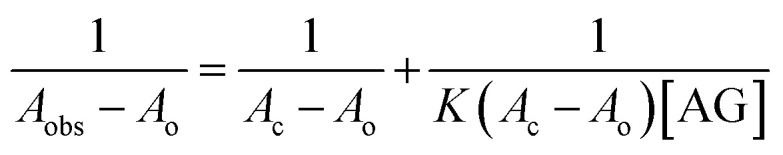
Here, *A*_obs_ is absorbance of HSA solution with variable concentrations of AG at 278 nm. *A*_o_ and *A*_c_ are the absorbance values for HSA and HSA–AG complexes. [AG] is the concentration of AG expressed in mol L^−1^. The *K* value was determined from a graphical plot (Fig. 3A inset) and that was found to be 4.76 × 10^4^ M^−1^.

The free energy of interaction was computed from binding constant *K* using the equation

4 Δ*G* = −*RT* ln *K*

where Δ*G* is the binding free energy, *R*, gas constant 1.987 cal mol^−1^ and *T* 310.15 K. The value of Δ*G* was found to be −6.6 kcal mol^−1^. The negative value of Δ*G* further confirmed the spontaneity of AG for HSA binding.

Fluorescence quenching study is a very sensitive and convenient technique for an insight into HSA molecular interactions. Intrinsic fluorescence property of HSA is selective due to a single tryptophan residue (TRP 214) occurring at the IIA subdomain (Sudlow's site I) of HSA.^28^ This is often attenuated upon interaction with a small molecule in its vicinity.^42^ HSA exhibited a characteristic emission peak at 350 nm upon its excitation at 280 nm. A gradual decrease of intensity of the characteristic emission band of HSA at 350 nm was recorded (Fig. 3B) with increasing concentration of AG. This confirmed the inclusion of AG into the binding site of HSA and subsequent resulted in formation of HSA–AG complex. However, no change of maximum emission wavelength was recorded, which suggested that the native protein conformation perhaps remained intact, even upon binding with AG.^43^

### 3.3 Molecular docking and stimulation

**Fig. 4:**
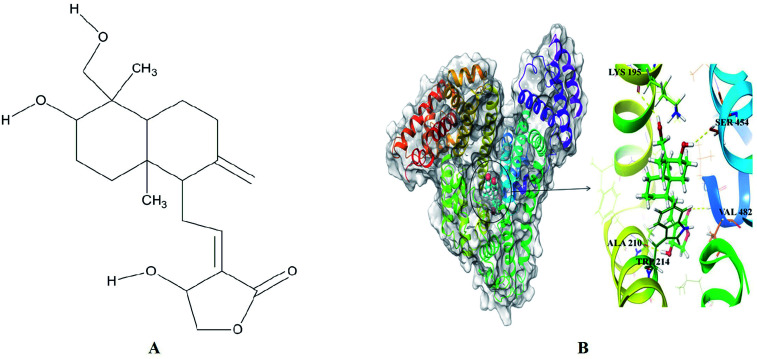
(A) Chemical structure of andrographolide (B) surface view of the crystal structure of HSA (PDB ID: 2BXP) bound to docked andrographolide showing H-bond (yellow dotted lines) interactions with binding site residue.

The best dock pose (Fig. 4B) showed the Glide XP and IFD score of −11.95 and −1299.82 kcal mol^−1^. Glide XP score indicated binding affinity of ligand towards protein target. IFD score is combination of Glide XP score and prime refinement energy of binding site. AG is a diterpenoid containing α-alkylidene γ-butyrolactone moiety with an allylic nature of hydroxyl group at the 14^th^ carbon position (Fig. 4A). AG extended hydrogen bond interactions with LYS 195, ALA 210, SER 454 and VAL 482 of HSA (Fig. 4B). Residues LYS 195 and SER 454 acts as H-bond acceptors for hydroxymethyl and hydroxyl groups of decahydronaphthalene ring occurring at a distance of 2.95 Å and 2.31 Å. Residue ALA 210 established H-bonding at a distance of 1.77 Å with the hydroxyl group of dihydrofuran ring, while carbonyl group from same ring formed H-bond of length 2.33 Å with VAL 482 residue. Apart from H-bond interactions decahydronaphthalene ring of andrographolide was also found interacting with TRP 214 through hydrophobic interactions. The molecular docking studies placed the perfect evidence in order to explain the quenching of intrinsic fluorescence signals at 350 nm. These site-specific interactions played a favorable role in stabilizing the native protein structure and thus validated the results obtained from UV-Vis and fluorescence spectroscopy. Molecular docking results corroborated perfectly with the wet lab experiments. Apparently, AG gets attached to the HSA binding cavity and concentration dependently destroyed hydrogen bonding networks. This was likely to cause to minor orientation changes without affecting much in protein conformation or helicity.

### 3.4 Effect of AG on HSA fibril morphology

**Fig. 5:**
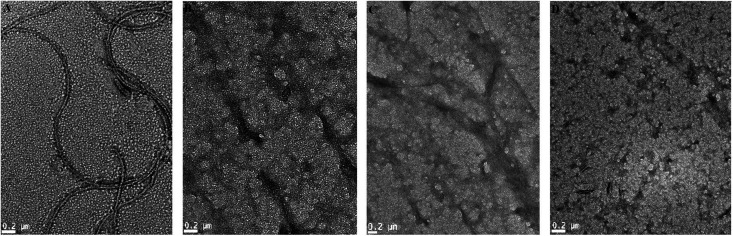
TEM images of 2 μM HSA solution incubated alone (A) and with AG at ratios of (B) 1 : 0.5, (C) 1 : 1, (D) 1 : 2. Scale: 0.2 μm.

Transmission electron microscopy experiments were performed in order to further investigate the effect of AG on morphologies of HSA aggregates. In absence of AG, HSA developed into long, un-branched and compact structures when incubated at 65 ± 2 °C for 6 days (Fig. 5A). The width of matured fibrils was found up to 30 nm. In contrast, samples treated with higher ratio of AG (1 : 2) formed disorganized amorphous aggregates rather than regular fibrils (Fig. 5D). HSA incubated with lower ratios of AG exhibited a tendency to form indistinct fibrillar aggregates (Fig. 5B and C), indicating that AG inhibited maturation of amyloid fibrils through stabilization of the protein structure at pre-fibrillar states.^44^ This was likely due to AG attachment in HSA IIA subdomain binding cavity and subsequent disruptions of hydrogen bonding networks. AG binding sites appeared specific which lead to minor unfolding of protein and the conformer resulting in hindered nucleation in fibrillation. The TEM experiments visually confirmed that the repressive effect of AG on HSA fibrillation is a function of concentration.

### 3.5 Effect of AG on HSA secondary structures

**Fig. 6:**
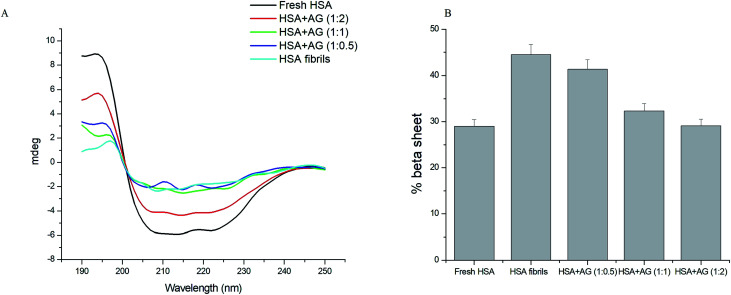
(A) Far UV CD spectra and (B) histograms of % β-sheet content in HSA solution in absence and presence of AG.

Far UV CD is a reliable tool to estimate the secondary structural changes of protein during fibrillation. Protein fibrillation associates progressive loss of structural integrity and β-sheets formations.^45^ Native HSA expressed 73% α-helix with characteristic CD spectrum featuring two negative bands at 208 nm and 222 nm (Fig. 6A). This corresponded with the typical α-helical contents of the unfolded HSA.^46^ HSA fibrillation led to loss of negative ellipticity at 208 and 222 nm due to destabilization of α-helix structures and concomitant sheet formations (44.5%). AG concentration dependently inhibited evolution of β-sheets formations. Extent of secondary structures formations were further quantified using online server BeStSel (http://www.bestsel.elte.hu).^47^ The β-sheet content relative percentage decrease was 27.4% when HSA was incubated with AG at 1 : 1, while that value was 34.6% upon co-incubation with AG at 1 : 2 (Fig. 6B). AG was substantially effective in inhibiting HSA fibrillation through stabilization of native protein structure and the results were in agreement with earlier spectroscopy observations.

## 4. Conclusion

The present study revealed that AG suppressed fibrillation of human serum albumin in concentration dependent manner. Findings indicated that AG stabilizes native protein tertiary structure and restricts protein fibril formations. CD studies established that AG stabilizes the HSA protein alpha helical structure prior hand. Site specific interactions for AG were noted in UV interaction studies, fluorescence spectroscopy and *in silico* experiments. The non-covalent interactions between AG and HSA significantly deterred evolution of fibrils. This work is particularly significant as there is minimal research available on bioactives demonstrating inhibitory action on fibril formations and protein tertiary structure stabilization.

## Conflicts of interest

Authors would also like to acknowledge that there is no conflict of interest in publication of this article.

## Supplementary Material
